# Glucagon-Producing Cell Expansion in Wistar Rats. Changes to Islet Architecture After Sleeve Gastrectomy

**DOI:** 10.1007/s11695-021-05264-6

**Published:** 2021-02-22

**Authors:** José Bancalero-delosReyes, Alonso Camacho-Ramírez, José Fernández-Vivero, Antonio Ribelles-García, Manuel Macías-Rodríguez, David Almorza-Gomar, Carmen Carrasco-Molinillo, María Ángeles Mayo-Ossorio, José-Arturo Prada-Oliveira, Gonzalo-Martín Perez-Arana

**Affiliations:** 1 Servicio Extremeño de Salud, Complejo Hospitalario de Badajoz, Badajoz, Spain; 2 Surgery Unit, Puerta del Mar University Hospital, University of Cadiz, Cadiz, Spain; 3 Asociación Gaditana de Apoyo al Investigador (AGAI), Cadiz, Spain; 4 Institute for Biomedical Science Research and Innovation (INIBICA), University of Cadiz, Cadiz, Spain; 5 Department of Human Anatomy and Embryology, Faculty of Medicine, University of Cadiz, Cadiz, Spain; 6 Sustainable Social Development Research Institute (INDESS), University of Cadiz, Cadiz, Spain; 7 Digestive Service, Puerta del Mar University Hospital, University of Cadiz, Cadiz, Spain; 8 Operative Statistic and Research Department, University of Cadiz, Cadiz, Spain

**Keywords:** Alpha-cells, Pancreas, Glucagon, Sleeve gastrectomy, Incretins, Cellular differentiation

## Abstract

**Purpose:**

Many studies about bariatric surgery have analyzed the effect of sleeve gastrectomy (SG) on glucose improvement, beta-cell mass, and islet size modification. The effects of SG on the other endocrine cells of the pancreas, such as the alpha-cell population, and their regulatory mechanisms remain less studied.

**Materials and Methods:**

We focused our work on the changes in the alpha-cell population after SG in a healthy model of Wistar rats. We measured alpha-cell mass, glucose tolerance, and insulin release after oral glucose tolerance tests and plasma glucagon secretion patterns after insulin infusion. Three Wistar rat groups were employed: SG-operated, surgical control (Sham), and fasting control.

**Results:**

The results obtained showed significant increases in the alpha-cell population after SG. The result was an increase in beta-cell transdifferentiation; it was shown by some expressed molecules (the loss of expression of Pdx-1 and the increase in Arx and Pax6 cells/mm^2^ of islet). The serum results were enhanced plasma glucagon secretion pattern after insulin infusion assays and normal glucose tolerance and insulin release after OGTT.

**Conclusion:**

We concluded that SG leads to an expansion of the alpha-cell population, at expense of beta-cell; this expansion of alpha-cells is related to transdifferentiation. Plasma glucose level was not affected due to an increased glucagon response.

## Introduction

Type 2 diabetes mellitus (T2DM) is an endocrine disease implicating several cell types within the islets of the pancreas. A large number of studies have focused on the beta-cell population and insulin production after bariatric procedures. Many of these studies focused on several hormones that were responsible for beta-cell changes. The effect of glucagon-like peptide 1 (GLP-1) or ghrelin on insulin production and the protective role of GLP-1 on beta-cell mass is well-known [1, 2]. In contrast, changes in the alpha-cell population and glucagon release have not been studied enough. Dysregulated glucagon secretion has been implicated in hyperglycemia in patients with type 2 diabetes [3]. A few studies have reported the effect of enterohormones stimulating alpha-cell secretion of glucagon, such as ghrelin [4], meanwhile GLP-1 suppressed this release [5].

Otherwise, the capability of alpha-cells to transdifferentiate into beta-cells after beta-cell depletion has also been reported [6], but this beta-cell to alpha-cell transdifferentiation plasticity implies long-term sustained hyperglycemia [7].

All these findings lead us to think about the changes in islet architecture, focusing on the alpha-cell component and, therefore, the role of alpha-cells on the maintenance of glucose homeostasis after an exceptionally stressful situation, such as bariatric surgery. In this landscape is the controversial role of oxidative stress factors after bariatric surgical procedures. Prior studies have shown that oxidative stress inhibits the activity of nuclear beta-cell transcription factors, such as Pdx-1. Pdx-1 is a factor that primes the loss of beta-cell identity, which induces alpha-cell genes [8].

Another interesting transcription factor is Pax6. Pax6 deletion implies the decrease of insulin production and glucagon gene expression. Pax6 is downregulated in diabetic db/db mice [9]. Paradoxically, the disruption of insulin signaling does not lead to alpha-cell hyperplasia in rodent models. This disruption of insulin signaling results in the transdifferentiation of a small subpopulation of alpha-cells to beta-cells [10, 11]. Recently, Aristaless-related gene homeobox (Arx) and DNA-methyltransferase 1 (Dnmt-1) have been reported as markers when alpha-cells decrease. These markers promote the conversion of alpha-cells to native beta-cells [12].

All of this seems to suggest a two-way path between beta-cell and alpha-cell populations. Thus, we found an interesting feature to study within the physiological modifications of the endocrine pancreas after bariatric surgery. We decided to reproduce the most common clinical bariatric surgery, the sleeve gastrectomy (SG). We employed a healthy Wistar rat model to analyze the pancreatic islet architecture and changes in the alpha-cell population. These changes in alpha-cells have been related to other pancreatic endocrine cells. We studied the glucose changes after SG, in order to clarify the role of alpha-cells in any glycemic component of the improvement in T2DM after bariatric surgery.

## Research Design and Methods

### Animals

Eighteen male Wistar rats, weighing 200–220 g and 10–11 weeks old, were provided and kept at the Experimentation and Animal Production Service of University (SEPA). All animal procedures were performed with the approval of the University Committee for the Ethical Use and Care of Experimental Animals. This committee ensured that the procedures in all experiments were performed in accordance with the international relevant guidelines and regulations of animal welfare.

Wistar rats were randomly divided into three groups: fasting control (FC) (*n* = 6), surgical control (sham-operated, Sham) (*n* = 6), and sleeve gastrectomy-operated (SG) (*n* = 6) (Fig. 1.1). After surgery, the animals were stable for 12 weeks before being sacrificed. The animals in the FG group (*n* = 6) received the same perioperative fasting periods related to the surgical protocol. The animals finished a 12-h presurgical and a 12-h postsurgical fasting period, respectively. An intake readaptation period followed the surgeries to normalize fasting. This stage was established for the FG and surgical groups. To evaluate the effect of bariatric surgery in animals, we observed the weight gain of the animals every 2 days, which was measured in grams (g).

**Fig. 1:**
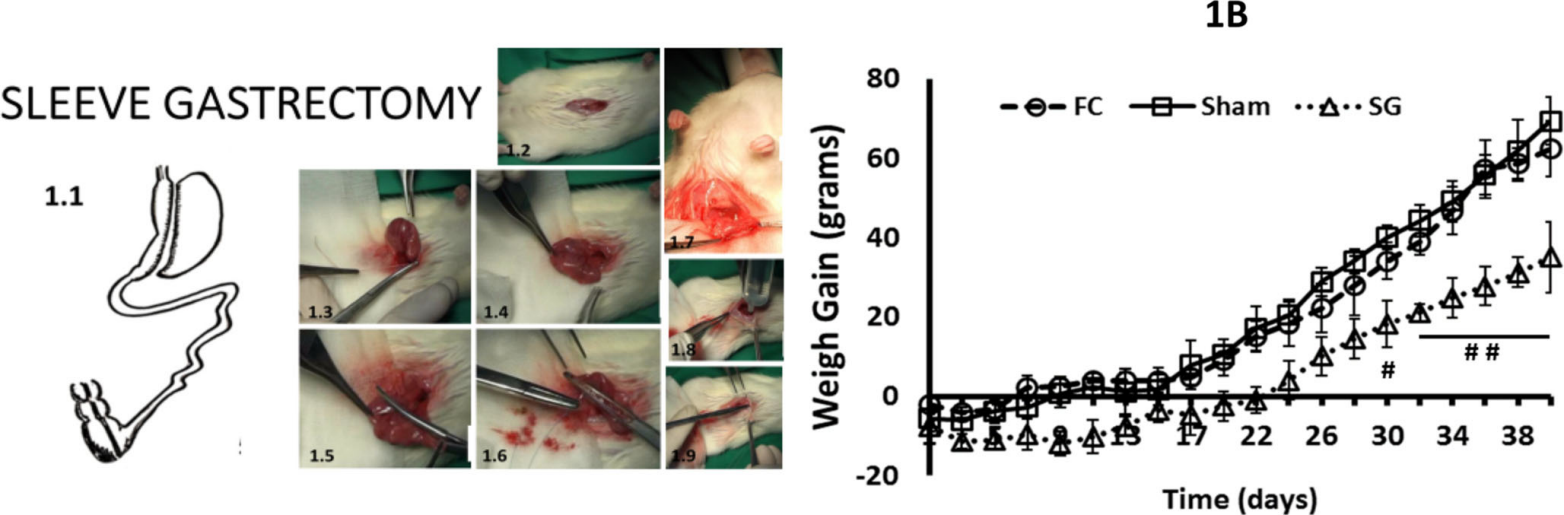
.2 Weight gain in fasting control (FC) rats (*n* = 6) (discontinuous black line with circles), sham-operated (Sham) rats (*n* = 6) (solid black line with squares), sleeve gastrectomy-operated (SG) rats (*n* = 6) (discontinuous black line with triangles) is presented on the Y axis, as grams (g). On the X axis, the survival period over 40 days is presented. Values are expressed as the mean ± SEM (# *P* < 0.05; ## *P* < 0.01)

### Surgical Procedures

Surgical procedures were performed in anesthetized animals with continuous inhalation of isoflurane 3% v/v (Isoflo, Abbott 571329.8). The sham technique (Sham) (*n* = 6) reproduced the surgical aggression on the intestinal tube but finally maintained its integrity. A laparotomy of 5 cm in the upper third of the abdomen, with an incision of approximately 3 cm in the middle area of the abdomen, was performed, exposing the small bowel. The jejunum was transected 40-cm distal to the angle of Treitz, and a terminus-terminus anastomosis was performed. The abdominal layers were closed as above. The abdominal muscular and skin layers were closed in one layer using a continuous suturing technique.

A laparotomy of 5 cm in the upper third of the abdomen (Fig. 1), through sectioning of the gastrosplenic ligament and exposing the stomach (Fig. 1.3, 1.4), was performed on the SG group (*n* = 6). Curved forceps were applied from the angle of Hiss to the antrum (Fig. 1.5), resulting in a cylindrical stomach approximately 0.5 cm in diameter. The stomach was sectioned and most of the fundus, corpus at the greater curvature, and antrum were resected (Fig. 1.6). The cardia and pylorus were preserved, as well as the lesser curvature (Fig. 1.7). In the SG group, the initial stomach volume was reduced by approximately 20%. Thus, we reproduced the actual selective technique used in humans. The abdominal layers were closed as above (Fig. 1.8, 1.9).

### Oral Glucose Tolerance Test, Insulin, and Glucagon Measurement

Four weeks after surgery, oral glucose tolerance tests (OGTTs) were performed in the FG, Sham, and SG groups. After a 12-h fasting period, 2 g/kg (20% v/v) glucose solution was administered through an oral gavage. Glucose levels were measured by glucometer (Glucocard G-Meter 1810, Menarini Diagnostics, Italy) in blood samples obtained from the tails of rats at 0, 30, 60, 90, and 120 min after administration of a glucose solution. The results were expressed as glucose mg/dl plasma.

Additionally, 4 weeks after surgery insulin was measured in blood samples from the tails of rats, every 15 min for 60 min, after administration of the glucose solution administration. For this, we used an ELISA kit (ALPCO Diagnostics, Salem, NH) according to the manufacturer’s instructions. The area under curve (AUC) was calculated by trapezoidal rule for every parameter in the study.

### Insulin Infusion Assays

Insulin infusion assays were performed 6 weeks after surgery in every rat group, with a 1.0 U/kg intraperitoneal insulin infusion. Glucose levels were measured in fasting rats in each group every 10 min for 60 min after an insulin infusion using a glucometer (Glucocard G-Meter 1810, Menarini Diagnostics, Italy) in blood samples obtained from the tails of rats. The results were expressed as glucose mg/dl plasma.

The plasma glucagon concentration was measured in fasting rats in each group every 10 min for 60 min after the insulin infusion. The plasma was removed from the blood samples obtained from the tails of rats, added to EDTA tubes and centrifuged at 4000 x g for 15 min at 4 °C. Total glucagon was assessed by an ELISA kit (Millipore-Merck, Merck KGaA, Darmstadt, Germany) according to the manufacturer’s instructions. The results were expressed as glucagon pg/ml plasma.

### Sacrifice and Pancreas Preparation

All animals were sacrificed 12 weeks after surgery by isoflurane inhalation overdose. The pancreases were immediately removed and fixed in Bouin’s solution overnight at 4 °C. Later, the samples were dehydrated, embedded in paraffin, and cut into serial 8-μm microtome sections.

### Pancreas Immunostaining

In rehydrated sections of pancreas, the beta- and alpha-cell populations were analyzed by immunostaining by using guinea pig anti-insulin Ig G antibody (Sigma-Aldrich, St. Louis MO, USA) and rabbit anti-glucagon IgG antibody (Abcam, Cambridge CB4 OFL, UK). The alpha-cell proliferation ratio was calculated by double immunostaining using anti-PCNA IgG antibody (Abcam, Cambridge CB4 OFL, UK) and anti-glucagon IgG antibody. The differentiation markers Arx, Pdx-1, and Pax6 were also analyzed using rabbit anti-Arx IgG, rabbit anti-PDX-1, and rabbit anti-Pax6 antibodies (Abcam, Cambridge CB4 OFL, UK). Fluorescent secondary antibodies Alexa 546 and 488 (Molecular Probes Inc. Eugene, OR, USA) were used against the primary antibodies. DAPI was used to counterstain the nuclei.

The alpha-cell proliferation ratio was quantified in 50 islets per condition. The results were noted under randomized conditions by a single investigator and expressed as number of glucagon PCNA+ cells and glucagon+ cells/mm^2^ of islet area.

Pax6-, Arx-, and Pdx-1-positive cell numbers were evaluated in four slices of whole pancreas per condition and expressed as number of Pax6-, Arx-, or PDX-1-positive cells/mm^2^ of pancreas.

Every histological parameter was measured and noted by a single investigator using a fluorescence microscope with digital camera and image analysis Cell-D software (Olympus, GmbH. Hamburg, Germany).

### Statistical Analyses

Data were presented as the means ± SEM. For AUC, histological, and weight gain data analysis, one-way ANOVA followed by Tukey’s/Bonferroni’s post hoc test was conducted using SPSS V21.0 software. Statistical significance was accepted at *P* < 0.05.

## Results

### Weight Gain

First, we measured weight gain in the FC, Sham, and SG groups for 40 days after surgery (Fig. 1B). There were no differences between any groups from the first day to the thirty fourth day, but statistically significant differences appeared between the FC and SG groups from the thirtieth day (*P* < 0.05) and from the thirty second day to the end of the survival period (*P* < 0.01).

### OGTT and Insulin Secretion

**Fig. 2:**
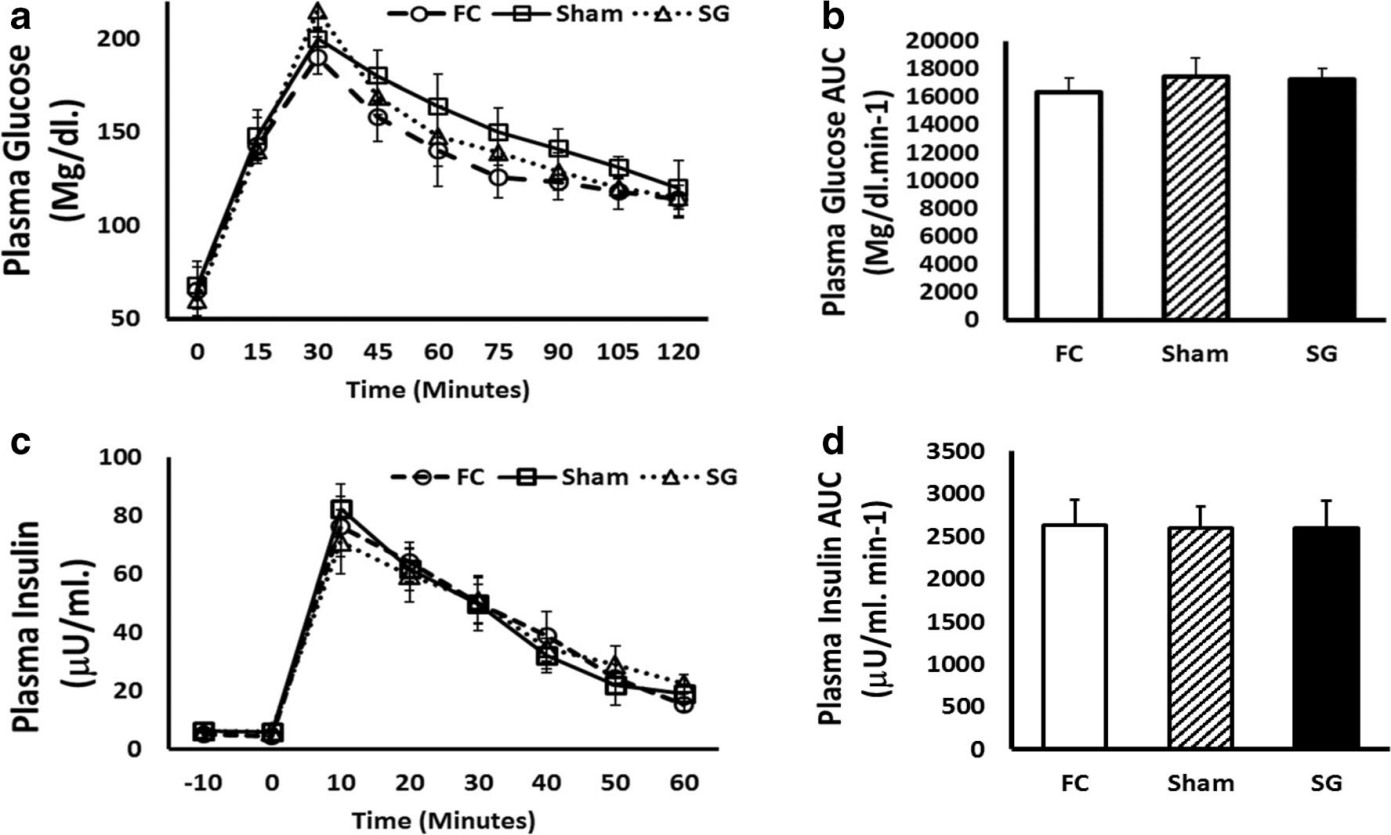
**a** Oral glucose tolerance tests (OGTTs) in fasting control (FC) rats (discontinuous black line with circles), sham-operated (Sham) rats (solid black line with squares), and sleeve gastrectomy-operated (SG) rats (discontinuous black line with triangles). Glucose levels are represented as mg/dl in the Y axis versus time after glucose ingestion in the X axis. Values are expressed as the mean ± SEM. **b** Area under curve (AUC) values are presented as mg/dl min^-1^ in the Y axis, expressed as the mean ± SEM (FC, 16312.5 ± 1007.53; Sham, 17430.0 ± 1360.89; SG, 17212.50 ± 780.49). **c** Plasma insulin in fasting control (FC) rats (discontinuous black line with circles), sham-operated (Sham) rats (solid black line with squares), and sleeve gastrectomy-operated (SG) rats (discontinuous black line with triangles). Plasma insulin levels are represented as μU/ml in the Y axis versus time after glucose ingestion in minutes in the X axis. Values were expressed as the mean ± SEM. **d** Area under curve (AUC) values were presented as μU/ml min^-1^ in the Y axis and expressed as the mean ± SEM (FC, 2631.50 ± 297.0; Sham, 2601.50 ± 247.50; SG, 2593 ± 322.05)

Four weeks after surgery, OGTTs were performed in the FC, Sham, and SG groups. Similar curves were described for the three groups. No significant differences between the glucose tolerance patterns of the FC, Sham, and SG groups were observed at any time (Fig. 2a). The AUC was calculated for the three groups, but no statistically significant differences were found between the groups (Fig. 2b). Normal glucose tolerance was confirmed in all groups 4 weeks after surgery.

Plasma insulin secretion was analyzed in the three groups 4 weeks after surgery. Similar plasma insulin secretion patterns were shown by the FC, Sham, and SG groups, but no statistically significant differences were noted (Fig. 2c). No statistically significant differences in the AUCs for plasma insulin appeared between the groups (Fig. 2d), which confirmed a normal plasma insulin secretion.

### Glucose and Glucagon Secretion Assay After Insulin Infusion

**Fig. 3:**
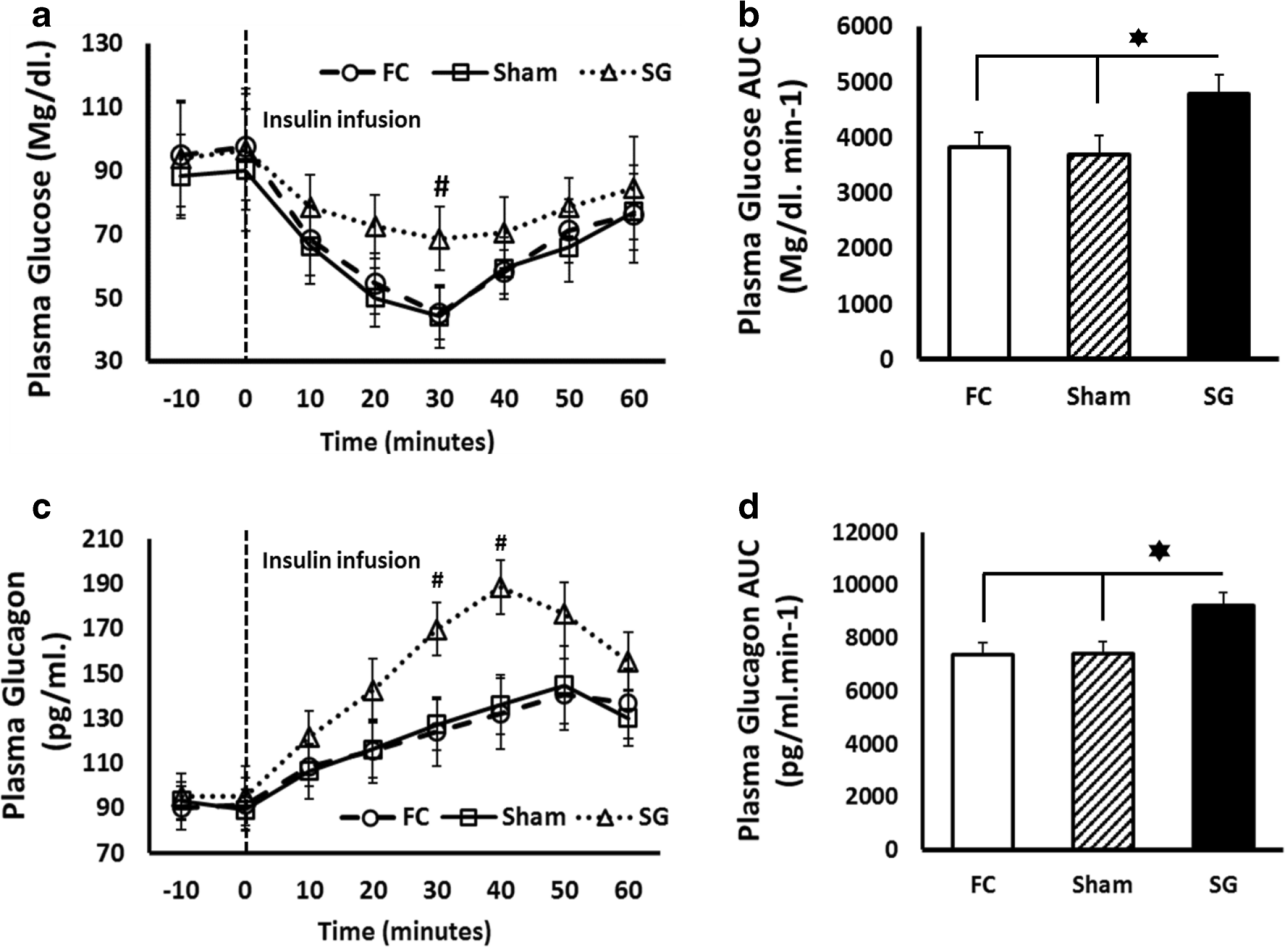
**a** Glucose plasma level after insulin administration in fasting control (FC) rats (discontinuous black line with circles), sham-operated (Sham) rats (solid black line with squares), and sleeve gastrectomy-operated (SG) rats (discontinuous black line with triangles). Glucose levels were represented as mg/dl in the Y axis versus time after insulin administration in the X axis. Values were expressed as the mean ± SEM (#*P* < 0.05). **b** Area under curve (AUC) values were presented as mg/dl min^-1^ in the Y axis and expressed as the mean ± SEM (*P < 0.05) (FC, 3833.5 ± 267.7; Sham, 3689.0 ± 344.0; SG, 4790.0 ± 338.65. **c** Glucagon plasma levels after insulin administration in fasting control (FC) rats (discontinuous black line with circles), sham-operated (Sham) rats (solid black line with squares), and sleeve gastrectomy-operated (SG) rats (discontinuous black line with triangles). Plasma glucagon levels were represented as pg/ml in the Y axis versus time after insulin administration in the X axis. Values were expressed as the mean ± SEM (#*P* < 0.05). **d** Plasma glucagon area under curve (AUC) values were presented as pg/ml min^-1^ in the Y axis and expressed as the mean ± SEM (*P < 0.05) (FC, 7347.5 ± 453.6; Sham, 7401.0 ± 447.0; SG, 9236.0 ± 494.90)

We also tested the existence of changes in glucose and glucagon plasma level patterns. These parameters were assayed after insulin infusion. As Fig. 3a shows, statistically significant differences in the plasma glucose pattern appeared between the SG and FC groups 30 min after insulin infusion (*P* < 0.05). We found a significant increase of plasma glucose secretion AUC in the SG group compared to the FC and Sham groups (*P* < 0.05) (Fig. 3b).

Figure 3c shows the plasma glucagon release pattern after insulin infusion. The plasma glucagon level of the SG group was significantly increased between 30 and 40 min after insulin infusion compared to the FC and Sham groups (*P* < 0.05). The plasma glucagon AUC of the SG group showed statistically significant differences compare to the FC and Sham groups (*P* < 0.05) (Fig. 3d).

### Histological Study

**Fig. 4:**
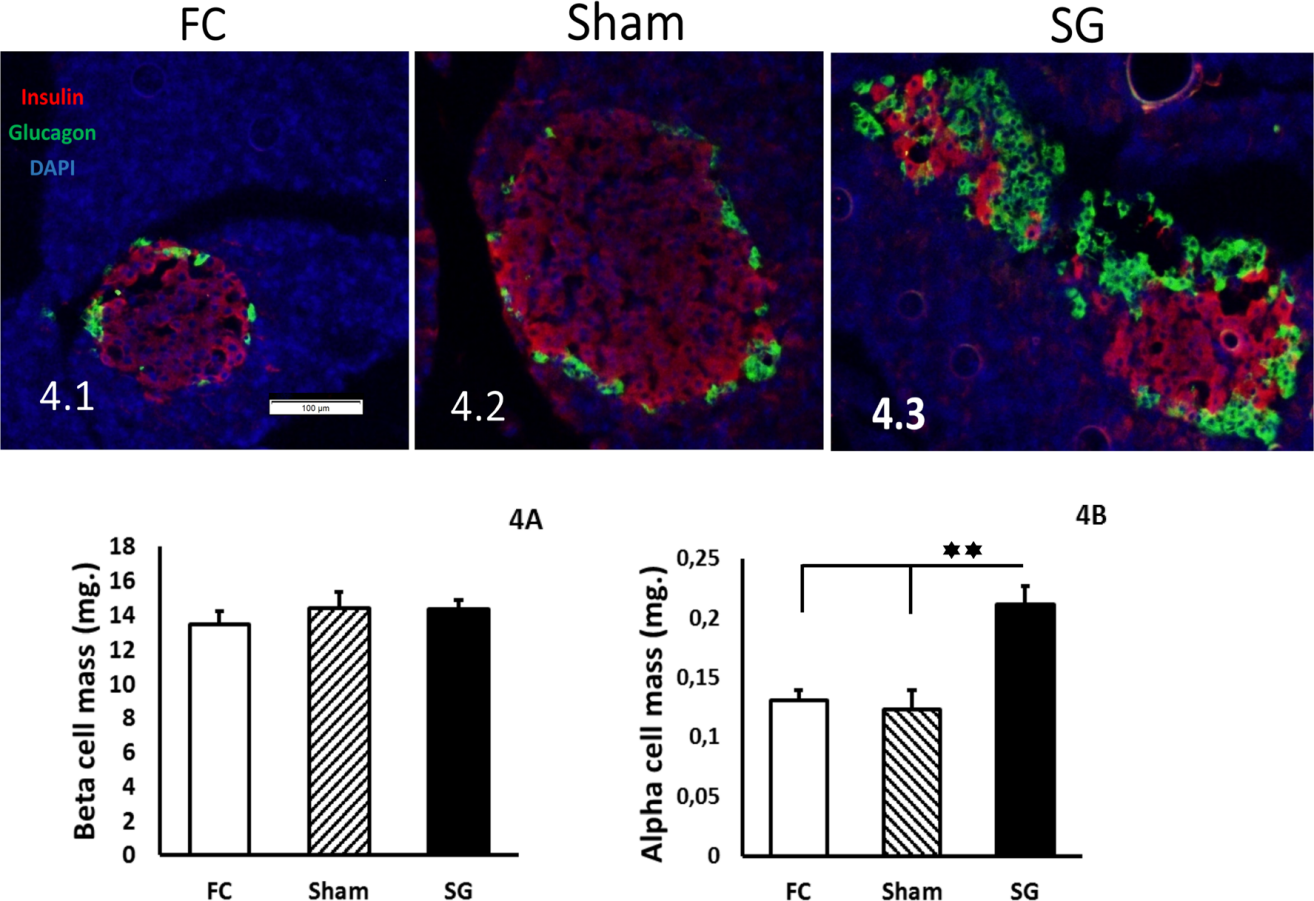
Beta-cell mass in green (Alexa 488); DAPI-counterstained nuclei in FC **(4.1)**, Sham **(4.2),** and SG **(4.3)** pancreas samples. **a** Beta-cell mass expressed in milligrams (mg) in the Y axis, as the mean ± SEM. Fasting control (FC) rats (white bar), sham-operated rats (Sham) (striped bar), and sleeve gastrectomy-operated (SG) rats (black bar). Data obtained were FC, 13.46 ± 0.75; Sham, 14.43 ± 0.94; and SG, 14.38 ± 0.52. **b** Alpha-cell mass expressed in milligrams (mg) in the Y axis as the mean ± SEM. (*P < 0.05; **P < 0.01). Fasting control (FC) rats (white bar), sham-operated (Sham) rats (striped bar), and sleeve gastrectomy-operated (SG) rats (black bar). Data obtained were FC, 0.130 ± 0.008; Sham, 0.123 ± 0.016; and SG, 0.211 ± 0.015

Twelve weeks after surgery, the beta- and alpha-cell masses were evaluated in pancreas samples from the FC, Sham, and SG rat groups. As Fig. 4a shows, no statistically significant differences appeared between the beta-cell mass values of the FC, Sham, and SG groups, but there were statistically significant differences in the alpha-cell mass values of the SG group compared to the FC and Sham groups (*P* < 0.05) (Fig. 4b).

### Alpha-Cell Proliferation Assay

**Fig. 5:**
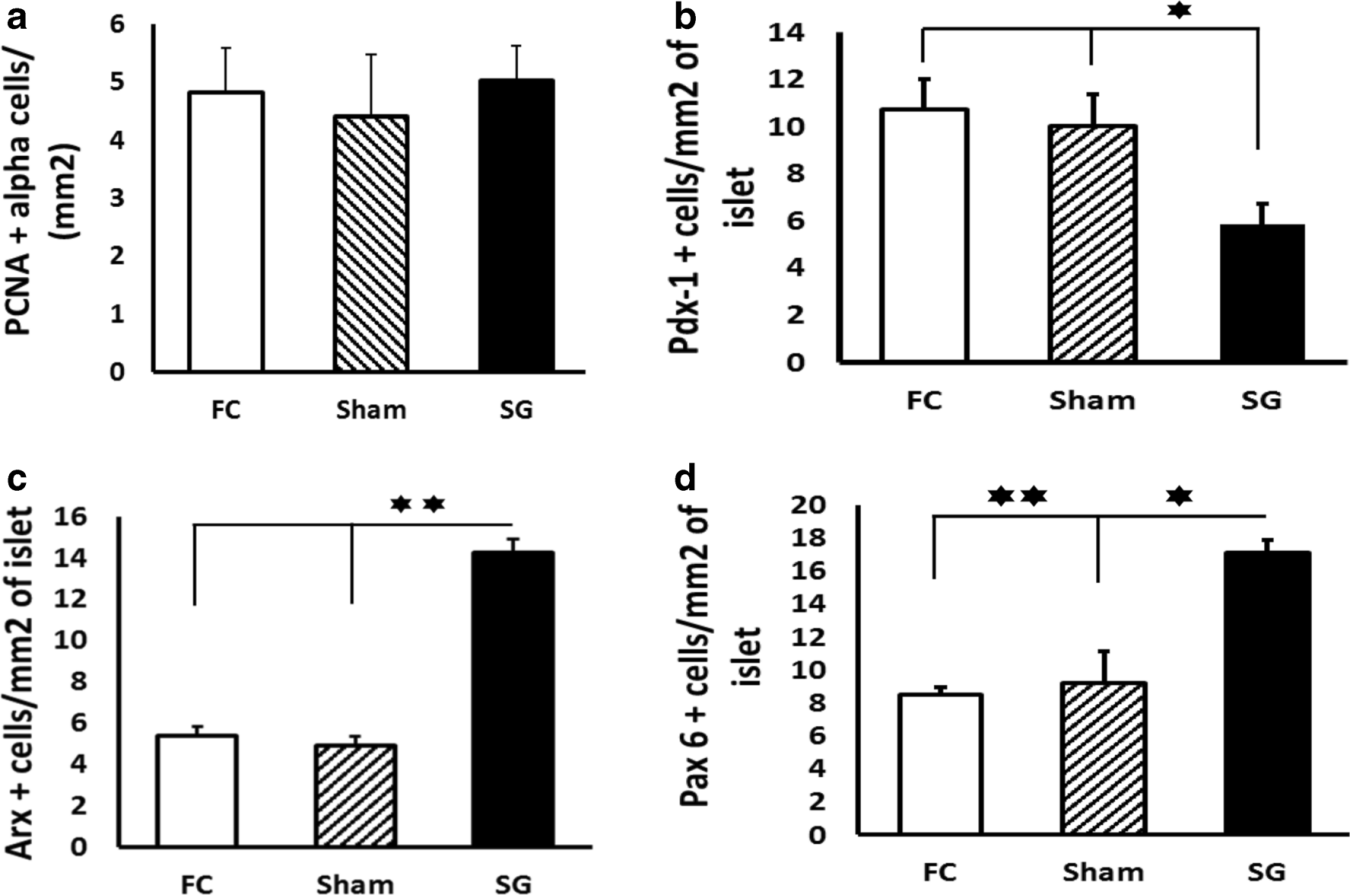
**a** Alpha-cell proliferation expressed as number of PCNA+ cells/mm^2^ of glucagon-positive area in the Y axis mean ± SEM. Fasting control (FC) rats (white bar); sham-operated (Sham) rats (striped bar); and sleeve gastrectomy-operated (SG) rats (black bar). Data obtained were FC, 4.81 ± 0.77; Sham, 4.40 ± 1.06; SG, 5.01 ± 0.60. **b** Pdx-1 expression in beta-cell area as Pdx-1+ cells/mm^2^ of insulin+ area in the Y axis, as the mean ± SEM. Fasting control (FC) rats (white bar); sham-operated (Sham) rats (striped bar); and sleeve gastrectomy-operated (SG) rats (black bar). **c** Arx expression in beta-cell area as Arx+ cells/mm^2^ of insulin+ area in the Y axis, as the mean ± SEM. Fasting control (FC) rats (white bar); sham-operated (Sham) rats (striped bar); and sleeve gastrectomy-operated (SG) rats (black bar). **d** Pax6 expression in beta-cell area as Pax6+ cells/mm^2^ of insulin+ area in the Y axis, as the mean ± SEM, in fasting control (FC) rats (white bar); sham-operated rats (Sham) (striped bar); and sleeve gastrectomy-operated (SG) rats (black bar)

No increase in the number of glucagon-secreting/PCNA-positive cells appeared in the SG group compared to the FC and Sham groups (Fig. 5a).

### PDX-1, Arx, and Pax6 Histological Study

Pdx-1 expression was analyzed in the pancreatic islet cells. Figure 5B shows low levels of Pdx-1-positive islet cells in the pancreas samples from the SG group compared with the samples from the FC and Sham groups (*P* < 0.05).

A significantly higher number of Arx-positive islet cells were found in the pancreas samples of the SG group but not in the pancreas samples of the FC and Sham groups (Fig. 5c).

The number of Pax6-positive islet cells was measured in the three groups (Fig. 5d). As Fig. 5c shows, an increased number of Pax6-positive cells were found in the FC and Sham groups compared to the SG group, with a statistically significant difference (*P* < 0.05).

## Discussion

Our intention was to probe the alpha-cell changes after SG. We followed three rat groups, two control groups, and one SG group. Therefore, functional changes around surgeries were measured before the sacrifice and pancreatic histological analyses. With regard to the functional tests, initially, there was a lower weight gain in the SG rats compared to both groups of control rats (Fig. 1). No effects on glucose tolerance, plasma insulin secretion, or OGTT were observed in SG rats (Fig. 2) compared to both groups of control rats. These data can be explained by a normal beta-cell mass, as it was found in the rats 12 weeks after bariatric surgery (Fig. 4a). As this study was performed in a healthy rat model, we must suppose a normal insulin sensibility at the liver.

We also explored changes in plasma glucose and glucagon profiles after insulin infusion in the SG and control groups 6 weeks after surgery. Surprisingly, significant differences were found in the recovery of normal glucose plasma levels in the SG rats after insulin infusion (Fig. 3a) compared to the control groups. There was no reason to presume changes in the liver insulin sensitivity of these healthy rats, so we only found an explanation. We supposed a retrieval of normal glucose with an increase in glucagon release after the insulin infusion test. Similar results were reported by Gudbrandsen et al. in humans, who found elevated plasma glucagon levels after SG [13]. To confirm this, we analyzed plasma glucagon profiles after insulin infusion. A higher curve was observed in the SG group compared to the FC and Sham groups, with an increase in the AUC of the SG group compared to the FC and Sham groups (Fig. 3b and c). These data confirmed a modified plasma glucagon release pattern in SG rats, which led us to think about a possible impact of SG surgery on the alpha-cell population.

The analysis of pancreas slices of the three groups indicated a statistically significant increase of alpha-cell mass in the SG group compared to the FC and Sham groups (*P* < 0.01) (Fig. 4b). This is supported by the plastic capacity of the alpha-cell population described in relation to different stress situations, such as pregnancy [14], fasting periods [15], or liver resistance to glucagon in mice [16]. The anatomical and functional rearrangements of the gastric tract after SG might hold the answer to this alpha-cell expansion. Supporting this idea, several published works reported increased populations of other pancreatic endocrine cells. Beta- or epsilon-cells have been reported to be increased in others after bariatric surgery, such as gastric bypass (RYGB) or even after SG, in rodents and humans [17–19].

We tried to clarify this element. We analyzed the alpha-cell proliferation ratio in SG rats and controls, but no statistically significant difference was found (Fig. 5a). These data led us to think about other possible expansion mechanisms for the alpha-cell population. These cellular mechanisms could be differentiation from ductal progenitors or transdifferentiation from other endocrine cell lineages. The roles of a large number of transcription factors in the maintenance of every islet endocrine cell phenotype are well-known. On the basis of this, a recent study proposed the conversion of beta-cells to alpha-cells in Fcor-knockout mice (Fcor KO). This was due to Fcor inhibition that drove an increase of Arx expression [20]. As a result, we examined the expression of Arx in the pancreas samples of the three study groups.

Surprisingly, a high expression of Arx was found in glucagon- and insulin-positive areas in the SG group compared to the FC and Sham group, as Fig. 5c shows (*P* < 0.01). This result supports our hypothesis about beta-cell to alpha-cell transdifferentiation after SG.

In addition, an increased number of Pax6-positive cells were also found in the pancreatic islets of the SG group compared to the FC and Sham groups (*P* < 0.05) (Fig. 5d). It is well-known that the Pax6 homeodomain is required for normal alpha-cell development [21] and glucagon gene expression [22]. This reinforces our hypothesis regarding beta-cell to alpha-cell transdifferentiation.

In opposition, no decrease in beta-cell mass was found (Fig. 4a). This paradoxical finding could be partially explained by the short survival period from surgery to sacrifice. Moreover, we focused on the results of the Pdx-1 transcription factor. Pancreatic duodenal homeobox-1 (Pdx-1) has been characterized as an insulin transcription factor, mostly restricted to beta-cells [8, 23]. In our findings, Pdx-1 showed low expression in insulin-positive area of islets in SG pancreas samples (*P* < 0.05) (Fig. 5b), which indicated beta-cell de-differentiation, although a high number of beta-cells could retain insulin granules in the cytoplasm. This would explain persistent insulin staining and a similar beta-cell mass value in the SG group compared to the FC and Sham groups.

On the other hand, a previous study has reported an increase in the pancreatic epsilon-cell population in healthy rats after SG [19]. This may be relevant in relation to the capacity of ghrelin to stimulate glucagon secretion in humans as reported by Broglio et al. [4]. This report was a previous consequence of our recent findings.

Taken together, all the data suggest a complex landscape in the pancreatic islets after SG. Thus, alpha-cell population expansion happens at the expense of the beta-cell population through a transdifferentiation mechanism after SG. However, preserved beta-cell mass allows for normal insulin secretion and glucose tolerance, with enhanced glucagon release in response to hypoglycemia. Thus, this whole mechanism supposes an integrated response to the functional changes in glucose absorption after SG.

However, a large number of questions remain around these findings, such as the exact mechanism that triggers these changes in the islet after surgery. Otherwise, we must expect that this transdifferentiation event can affect other islet endocrine populations, such as delta-cells. We must clarify how this process affects long-term beta-cell mass and function. All these questions should be studied in the future.

## Data Availability

All the data supporting the results and critical resources will be available at the institutional repository of the University of Cadiz (http://hdl.handle.net/10498/24086).

## Declarations

### Statement of Animal Welfare

The authors declare that ethical approval was obtained for our study. All procedures performed in studies involving animals were in accordance with the ethical standards of the institution at which the studies were conducted, and ethical approval was obtained from the University of Cadiz Committee for the Ethical Use and Care of Experimental Animals. This committee ensured that the procedures in all experiments were performed according to international guidelines and regulations for the welfare of the animals. The internal Committee for the Ethical Use and Care of Experimental Animals followed the instructions marked for the Autonomous Andalusian Authority.
